# Supplementary material for: Identification of photoperiod-induced specific miRNAs in the adrenal glands of Sunite sheep (Ovis aries)
Source: Front Vet Sci. 2022 Jul 22;9:888207. doi: 10.3389/fvets.2022.888207 (PMC9354845; doi:10.3389/fvets.2022.888207)
Supplement: Supplementary file 1 [file Data_Sheet_1.ZIP › Supplementary file/miRDeep2 file.zip!/miRDeep2 file/Know_Structure/SPLP42A2/oar-let-7c.pdf]

miRBase precursor : oar-let-7c
Total read count : 133209
oar-let-7c read count : 133141
remaining reads : 68

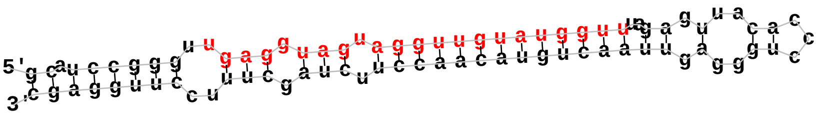

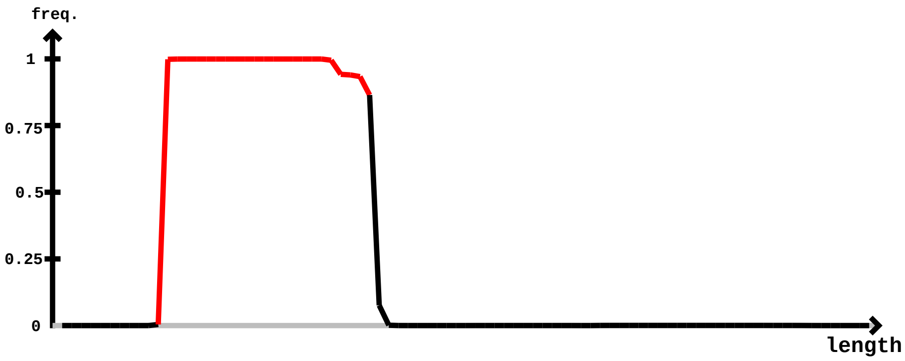

oar-let-7c

```
5'- gcauccggguugagguaguagguuguaugguuuagaguuacacccugggaguuaacuguacaaccuucuagcuuuccuuggagc   -3'    exp
    ((.((((((..(((.(((.(((((((((((((..((.(..((...))..).))))))))))))))).))).)))..))))))))    reads      mm         sample
    ........guugagguaguagguuguauAgu.....................................................    1          1          seq
    ........guugagguaguagguuguauggu.....................................................    2          0          seq
    .........uugagguaguagguuguaug.......................................................    2          0          seq
    .........uugagguaguagguuguaugg......................................................    13         0          seq
    .........uugagguaguagguuguauggu.....................................................    162        0          seq
    .........uugagguaguagguuguauAgu.....................................................    2          1          seq
    .........uugagguaguagguuguauAguu....................................................    8          1          seq
    .........uugagguaguagguuguaugguu....................................................    352        0          seq
    .........uugagguaguagguuguaugguuu...................................................    20         0          seq
    .........uugagguaguagguuguauAguuu...................................................    2          1          seq
    ..........ugagguUguagguugua.........................................................    1          1          seq
    ..........ugagguagGagguugua.........................................................    84         1          seq
    ..........ugaAguaguagguugua.........................................................    4          1          seq
    ..........ugagguaguagguugua.........................................................    500        0          seq
    ..........ugagguaguagguuUua.........................................................    4          1          seq
    ..........ugaggCaguagguugua.........................................................    1          1          seq
    ..........ugagguaguaAguugua.........................................................    11         1          seq
    ..........ugagguaguaAguuguau........................................................    44         1          seq
    ..........ugaAguaguagguuguau........................................................    42         1          seq
    ..........ugagguaguagguuguau........................................................    6310       0          seq
    ..........ugagguaguagguuUuau........................................................    12         1          seq
    ..........ugagguagGagguuguau........................................................    619        1          seq
    ..........ugagguUguagguuguau........................................................    5          1          seq
    ..........ugagguaguagguuguaug.......................................................    300        0          seq
    ..........ugagguaguagguuguaugg......................................................    837        0          seq
    ..........ugagguaguagguuguauggu.....................................................    9017       0          seq
    ..........ugagguaguagguuguaugguu....................................................    104572     0          seq
    ..........ugagguaguagguuguaugguuu...................................................    9895       0          seq
    ..........ugagguaguagguuguaugguuua..................................................    54         0          seq
    ..........ugagguaguagguuguauAguuua..................................................    116        1          seq
    ..........ugagguaguagguuguauAguuuag.................................................    1          1          seq
    ..........ugagguaguagguuguaugguuuag.................................................    1          0          seq
    ...........gagguaguaAguuguau........................................................    1          1          seq
    ...........gagguaguagguuguau........................................................    5          0          seq
    ...........gagguagGagguuguau........................................................    1          1          seq
```

oar-let-7c

gcauccggguugagguaguagguuguaugguuuagaguuacacccugggaguuaacuguacaaccuucuagcuuuccuuggagc			
...........gagguaguagguuguauggu...	11	0	seq
...........gagguaguagguuguaugguu..	112	0	seq
...........gagguaguagguuguaugguuu...	10	0	seq
............agguaguagguuguaugguu..	4	0	seq
............agguaguagguuguaugguuu...	2	0	seq
..............guaguagguuguaugguu..	1	0	seq
................................uagaguuacacccugggag.................................	1	0	seq
................................uagaguuacacccugggagu................................	1	0	seq
................................uagaguuacacccugggaguu...............................	3	0	seq
................................uagaguuacacccugggaguuaa.............................	1	0	seq
....cccugggaguuaacuguacaaccu..................	1	0	seq
....uuaacuguacaaccuucuagcu...........	2	0	seq
....aacuguacaaccuucuagcuuu.........	1	0	seq
....cuguacaaccuucuagcuuu.........	2	0	seq
....cuguacaaccuucuagcuuuc........	12	0	seq
....cuguacaaccuucuagcuuucc.......	34	0	seq
....cuguacaaccuucuagcuuuccu......	5	0	seq
....uguacaaccuucuagcuuuccu......	5	0	seq
